# Direct to implant breast reconstruction by using SERI®, preliminary report

**DOI:** 10.1186/s13046-014-0078-5

**Published:** 2014-11-25

**Authors:** Roy De Vita, Ernesto Maria Buccheri, Marcello Pozzi, Giovanni Zoccali

**Affiliations:** Plastic Surgery Department, Regina Elena Cancer Institute of Rome, Rome, Italy

## Abstract

**Background:**

There has been a “rising tide” in mastectomy utilization that can be attributed to more skin-sparing mastectomies (SSMs) performed concurrently with immediate breast reconstruction. We report our experience of the first use of SERI® Surgical Scaffold (SERI®; Allergan, Inc.) in 21 cases of direct to implant (DTI) breast reconstruction after SSM.

**Methods:**

Our retrospective experience, from April 2013 to May 2014, is based on 21 cases of direct to implant (DTI) breast reconstruction after SSM (9 monolateral 6 bilateral). All the patients were oncological with a preoperative cancer stage was into 0–2 stage. In order to assess the level of satisfaction with the aesthetical result, on 4–13 months post-operative patients were asked to complete a questionnaire that evaluated various parameters by means of a Visual Analogue Scale (V.A.S.).

**Results:**

Over a 13-months period, a total of 15 patients underwent 21 immediate breast reconstructive procedures with Allergan Natrelle 410 style implants plus SERI® after SSMs. Definitive histological examination give evidence of 5 patients intraductal carcinoma, 6 patients multifocal carcinoma and 4 patients carcinoma in situ. 6 bilateral cases of direct to implant (DTI) breast reconstruction after SSM had a monolateral oncological treatment and on the other side a prophylactic treatment. At the end of the short follow up (minimum 6 months) all the patient were cancer free with an excellent outcome. Complication rate presents just one implant exposure followed by a revised surgery. At V.A.S. the mean patient satisfaction was 5,77 (good), 4,09 (fair) for sensitivity of the nipple areola complex, 6,33 (good) assessment of implant position, 6,28 (good) self esteem, 5,2 (good) attraction ability, 4,99 (fair) intimate life, 6,81 (good) overall feelings about breast reconstruction, 6,71 (good) simmetry.

**Conclusions:**

The really encouraging results of our early experience will help surgeons introducing SERI® into their practice to select appropriate patients for direct-to-implant single-stage immediate breast reconstruction. A larger study cohort and longer follow-up times are required to identify additional predictors and indications.

## Introduction

Mastectomy remains a common form of treatment for breast cancer [1,2]. In addition, there has been a “rising tide” in mastectomy utilization that can be attributed to more skin-sparing mastectomies (SSMs) performed concurrently with immediate breast reconstruction. This rise may be attributed to better identification of women at high risk for breast cancer with genetic testing, more refined methods of imaging, and a clearer picture of the late adverse effects of breast irradiation [1-4]. Immediate breast reconstruction has proven to be a safe and beneficial treatment for women diagnosed with early-stage breast cancer, and offers the benefits of improved body image, health-related quality of life (HRQOL), and patient satisfaction [5-12]. For women who have the option of undergoing breast conserving therapy or mastectomy, the selection of SSM with immediate reconstruction is preferred by those who want to avoid radiation and local recurrence, but do not wish to live with a mastectomy defect [13].

We report our experience of the first use of SERI® Surgical Scaffold (SERI®; Allergan, Inc.) in 15 patients, 21 cases of direct to implant (DTI) breast reconstruction after SSM. SERI® is the first silk derived bioresorbable scaffold (SBS), it is devoid of animal or human tissue and it is properly designed for breast reconstruction. We evaluated the complication rate, clinical course and postoperative outcomes by using a Visual Analogue Scale (VAS) of patients who underwent direct-to-implant breast reconstructions by using SERI and definitive breast implant.

## Material and methods

Our retrospective experience, from April 2013 to May 2014, is based on 21 cases of direct to implant (DTI) breast reconstruction after SSM (9 monolateral 6 bilateral). All the breasts were medium size. All the patients were oncological with a preoperative cancer stage was into 0–2 stage [14]. The exclusion criteria for the current study were as follows: 1) a history of undergoing irradiation before and after reconstruction, 2) a history of undergoing neoadjuvant chemotherapy as part of breast cancer treatment, and 3) a history of undergoing reconstructive surgery. All the patients were submitted to retroareolar ductal extemporary histological exam to confirm absence of cancer involvement of the NAC. So all the subcutaneous mastectomy were SSM [15,16]. Patients were followed up for a mean period ranging from 6 months to 12 months. Data on age, body mass index, mastectomy weight, duration of surgical drainage, cancer stage, presence of comorbidities including diabetes, hypertension, cardiac disease, and smoking history were tabulated (Table 1).

**Table 1:** **Patients characteristics**

Nr. of patients	15
Nr. of breasts treated	21
Median age (41–62)	51,5
Median B.M.I. kg/m^2^ (18,8-25,9)	22,3
Nr. Breast cancers stage 0 (Ca. in situ)	4 (26,6%)
Nr. Breast cancers stage 1 (Ca multifocal)	6 (40%)
Nr. Breast cancers stage 2 (Ca Intraductal)	5 (33,3%)
Number of active smokers	3 (14,2%)
Number of diabetes mellitus patients	1 (6,6%)
Number of hypertension patients	1 (6,6%)
Follow-Up months (range)	4-13 (9,5)
Duration of subcutaneous drainage, days	4-8 (6)
Duration of intra-pocket drainage, days	9-18 (13,5)

We evaluated patients characteristics, the clinical course and postoperative outcomes based on the following factors: 1) seroma formation, 2) infection, 3) hematoma, 4) skin flap necrosis, nipple areola complex (NAC) necrosis, 5) capsular contracture (Baker grade III or IV), 6) loss of implant.

All the surgical incision were located at the infra mammary fold laterally, SSM were performed by using Metzenbaun scissors to minimize seroma formation [9,15]. After SSM, during the same operation, the surgical technique utilized entailed submuscular dissection of the pectoralis major muscle, suturing of the SERI® to the inframammary fold, caudal edge of the pectoralis muscle, and the serratus anterior muscle laterally with 2–0 Vicryl interrupted suture, thus enveloping the implant with SERI® for inferior/lateral pole coverage. Two drains, one above the pectoralis major muscle and one in the pocket, were used on all patients. So the implant was inserted into the newly created submuscular pocket. The implant was covered superiorly with the pectoralis muscle and inferolaterally with a SERI® sling without arising serratus muscle and fascia. Prophylactic intravenous 2 gr Cefazolin antibiotic use and intraoperative antibiotic irrigation with riphampicine solution for implants was recorded for all patients. Daily antibiotic administration was in state untill drainges removed. All patients were discharged with 2 days hospitalization. Drains were removed when their output was <30 cc per 24 h for at least 2 consecutive days.

Moreover, in order to assess the level of satisfaction with the aesthetical result, on 6–13 months post-operatively, patients were asked without prior notice to complete a questionnaire that evaluated various parameters (Sensitivity of the NAC, Assessment of implant position, Self esteem, Attraction ability, Intimate life, Overall feelings about breast reconstruction, simmetry) by means of a Visual Analogue Scale (V.A.S.) [17]. Patients were instructed to use a numerical scale of 1 to 10, with one as the worst outcome and 10 as the best possible outcome (<5 = fair, 5 to 6.9 = good, and ≥7 = very good).

This study was approved by the Regina Elena National Cancer Institute review board and followed the principles of the Declaration of Helsinki and subsequent amendments.

All participants provided written informed consent before participating in the study.

## Results

Over a 13-months period, a total of 15 patients underwent 21 immediate breast reconstructive procedures with Allergan Natrelle 410 style implants assisted by SERI® for inferior/lateral pole coverage. Definitive histological examination give evidence of 5 patients intraductal carcinoma, 6 patients multifocal carcinoma and 4 patients carcinoma in situ. 6 bilateral cases of direct to implant (DTI) breast reconstruction after SSM had a monolateral oncological treatment and on the other side a prophylactic treatment [18]. At the end of the short follow up (minimum 4 months) all the patient were cancer free. Definitive implants in monolateral DTI breast reconstruction were: FF 375 (3) - 425 (1) - FF 475 (1), MF 335 (1) - 375 (2), LF 310 (1); two of nine monolaeral breast reconstruction needed contralateral breast reshaping by mastopexy to improve simmetry. 6 bilateral DTI breast reconstruction were performed by using FF 375 (1 pt)- 425 (1 pt), MF 420 (3 pts), MF 375 (1 pt). The Senior Author performed both procedures, SSM and reconstructive procedures at Regina Elena Cancer Institute, Plastic Surgery Department, using a standardized technique, above described, for subcutaneous mastectomy and DTI breast reconstruction with SERI® as a pectoral extender. The Senior Author performed as well the contralateral mastopexy in 2 cases as breast simmetrization. The encouraging preliminary results are showed in Table 2. Regarding the unique case of partial flap necrosis (4,7%), in an active smoker, monolateral breast reconstruction and subsequent implant exposure, the Senior Author performed a latissimus dorsi flap with implant substitution (Allergan Natrelle 410 Style FF 290) as a salvage with an excellent outcome.

**Table 2:** **Results**

Nr. Seroma formation self-limiting	1 (4,7%)
Nr. Late Seroma formation	0 (0%)
Infection	0 (0%)
Hematoma self-limiting	1 (4,7%)
Hematoma requiring surgical revision	0 (0%)
Partial skin flap necrosis	1 (4,7%)
NAC necrosis	0 (0%)
Capsular contracture	0 (0%)
Loss of implant	1 (4,7%)

**Figure 1:**
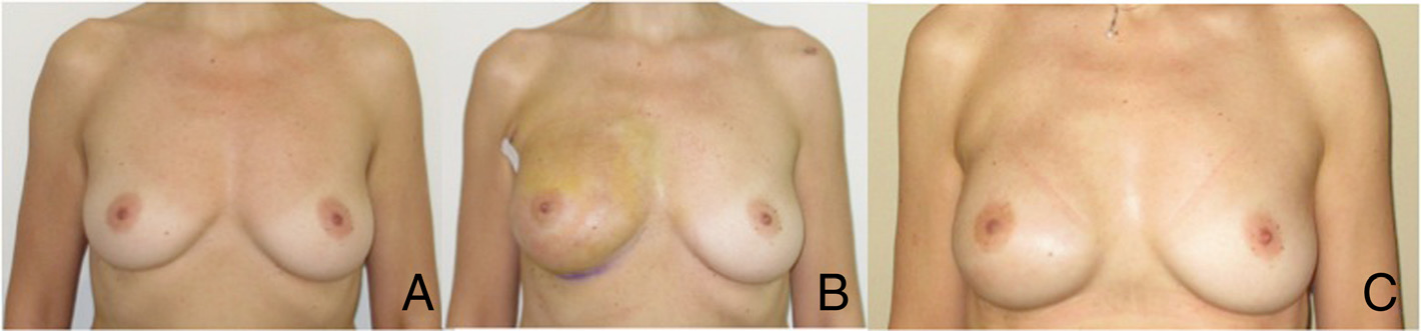
**Case 1. A**: 39 YO nulliparous, preoperative view, grade 1 intraductal carcinoma on the right breast. **B**: Right breast: therapeutic nipple skin sparing mastectomy direct to implant Allergan 410 Style FF 425 and SERI®. 3 weeks postop. **C**: 5 months postop.

**Figure 2:**
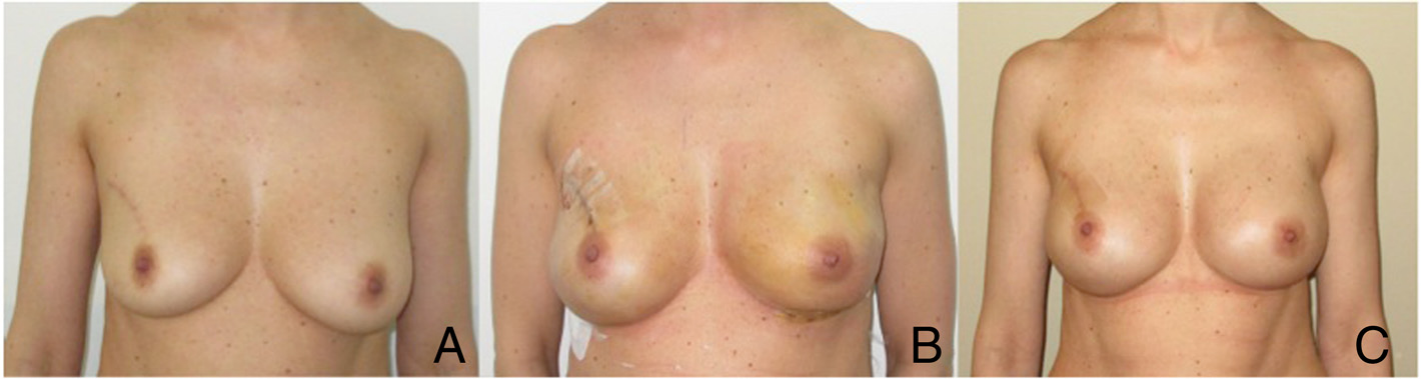
**Case 2. A**: 45 YO, preoperative view, previous quadrantectomy without Radiotherapy on the right breast, grade 1 multifocal carcinoma on the left breast. **B**: Left breast: therapeutic nipple skin sparing mastectomy direct to implant Allergan 410 Style MF 420 and SERI®. 2 weeks postop. Right breast: prophylactic nipple skin sparing mastectomy direct to implant Allergan 410 Style MF 420 and SERI®. 2 weeks postop. **C**: 4 months postop.

**Figure 3:**
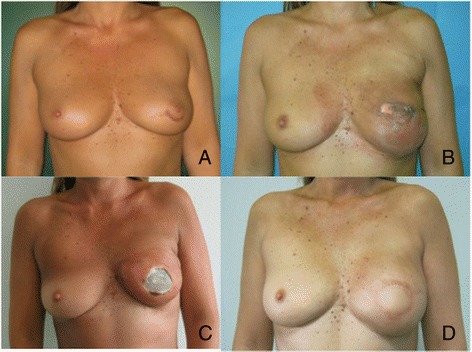
**Case 3. A**: 51 YO, preoperative view, previous biopsy grade 2 intraductal carcinoma on the left breast. **B**: Therapeutic nipple skin sparing mastectomy direct to implant Allergan 410 Style MF 375 and SERI®. 3 weeks postop, initial skin suffering. **C**: 2 months postop implant and SERI® exposure before reoperation. **D**: 7 months postop after implant explantation and Latissimus dorsi miocutaneous flap as a salvage procedure with Allergan 410 MF 335.

The mean patient satisfaction was 5,77 (good), 4,09 (fair) for sensitivity of the nipple areola complex, 6,33 (good) assessment of implant position, 6,28 (good) self esteem, 5,2 (good) attraction ability, 4,99 (fair) intimate life, 6,81 (good) overall feelings about breast reconstruction, 6,71 (good) simmetry (Table 3) (Figures 1, 2 and 3).

**Table 3:** **Visual Analogue Scale (VAS) administered to patients at 6–13 months follow-up. Mean, range of 15 patients**

**Patients parameter evaluation**	**Satisfaction rate**
Sensitivity of the nipple areola complex	4,09 (fair)
Assessment of implant position	6,33 (good)
Self esteem	6,28 (good)
Attraction ability	5,2 (good)
Intimate life	4,99 (fair)
Overall feelings about breast reconstruction	6,81 (good)
Simmetry	6,71 (good)
Mean patient satisfaction	5,77 (good)

## Discussion

Traditional prosthetic breast reconstruction consisting of a two-stage tissue expander-implant procedure is the most widely practiced technique in postmastectomy breast reconstruction. Disadvantages of this technique include two operations, multiple office visits for expansion, pain after expansion, and significant capsular adjustment at the time of implant exchange. Despite these disadvantages, this technique is safe and predictable and allows for good aesthetic results [19,20]. Acellular dermal matrices were introduced into clinical practice to aid in a variety of surgical problems, including complex abdominal wall reconstruction [21], facial paralysis [22], and dural defects [23]. In 2001, Duncan reported the first use of acellular dermal matrix in breast surgery to correct persistent implant rippling in both aesthetic and reconstructive cases [24]. In 2006, Salzberg reported experience using acellular dermal matrix to achieve a single-stage implant reconstruction with full implant coverage [25]. Since then, various types of tissue products have been developed for two stages and direct to implant breast reconstruction and these include AlloDerm (LifeCell Corp.), Strattice (LifeCell Corp.), DermaMatrix (Synthes Inc., West Chester, PA, USA), FlexHD (Ethicon Inc., Somerville, NJ, USA), and Permacol (Covidien, Mansfield, MA, USA) [25,26]. In addition, these products vary in many ways, depending on the source of tissue material, manufacturing methods, storage and surgical preparation, available size, and cost. However, their use is often limited due to the lack of outcome data [26,27]. Moreover the most recent major retrospective studies have demonstrated an high rates of complications such as infection, seroma formation, capsular contracture, implant exposure that needs reoperation with the above matrices.

In April 2013 Allergan presents and launches SERI® in the European and US market. It is the first silk derived bioresorbable scaffold (SBS) properly designed for breast reconstruction, BIOSILK™ is devoid of animal or human tissue. It is biocompatible which allows interaction with the body, it is bioresorbed by enzymatic and cellular digestion. Native tissue generation occurs in its place and bioresorption is expected within 18–24 months.

The porous design of SERI® allows for rapid neovascularisation and growth of functional tissue, gradual transfer from SERI® to new and well vascularised tissue provides consistent and predictable support in direct to implant single stage breast reconstruction.

BIOSILK™ purification removes impurities leaving an ultrapure bioprotein designed to provide an improved and consistent user experience. Operatively SERI® can be cut without unravelling, is easy to suture (large pores), does not require refrigeration or rehydration as the surgical ADM scaffold previously described [27,28]. Our experience reported start on April 2013 until May 2014, it is based on 21 cases of direct to implant (DTI) breast reconstruction after SSM (9 monolateral 6 bilateral) by using SERI as surgical scaffold to envelope the implant for inferior/lateral pole coverage. We had strictly restricted surgical incications in our series patients. All the breasts were medium size. All the patients were oncological with a preoperative cancer into 0–2 stage [15]. The patients enrolled doesn’t has a history of undergoing irradiation before and after reconstruction and neoadjuvant chemotherapy as part of breast cancer treatment.

Direct-to-implant single-stage immediate breast reconstruction has gained significant popularity as an elegant single-stage solution to postmastectomy prosthetic breast reconstruction [29-31]. In addition to completing breast reconstruction in a single stage, the benefits of SERI® include improved control of the breast pocket, augmentation of soft-tissue coverage in the lower pole, and a postulated decrease in capsular contracture [32-34]. In Litterature, one of the most common reasons for early revision in the direct-to-implant single-stage immediate breast reconstruction group is Baker grade III- IV capsular contracture (mean 34.6 percent of failed single-stage) [14]. Despite the postulated hypothesis that SERI® may prevent or alleviate capsular contracture, confirmed by our results, capsular contracture still remains one of the most likely causes for revision; so we need a further follow up to declare that DTI breast reconstruction by using SERI® and definitive implant present a low rate of capsular contracture.

Single-stage prosthetic breast reconstruction using surgical scaffold has widespread implications for patients and the health care system. Knowing the expense of surgical scaffold, cost analysis at our Institution showed that breast reconstruction with SERI® was cost effective compared with traditional two-stage reconstruction when performed in a single stage [35]. It has the potential to decrease surgical morbidity, surgical wait times, and operative costs. Moreover, it is important to accurately identify patients who are likely to have successful outcomes following direct-to-implant single-stage immediate breast reconstruction. An intraoperative algorithm based on flap thickness and quality of skin has been suggested to guide use of this technique [36]. It has also been advised that immediate implant-based reconstruction with or without the use of surgical scaffolds should be used with caution in large, ptotic breasts and in patients with a history of irradiation or chemotherapy [37]. Preoperative patient selection criteria specific to direct-to-implant single-stage immediate breast reconstruction using surgical scaffold do not exist in the Literature [25,26]; so our experience was to used the basic principle of reasoning: small, medium breasts leaving out potential risk factors such as Radiotherapy and Chemoterapy or heavy levels of comorbidities.

Thus, in this study, we analyzed our direct-to-implant single-stage immediate cohort and found that patients with small, medium breasts (A cup, 300-g mean mastectomy weight) were less likely to require an early surgical revision compared with Litterature data. Our guidelines allows us a 0% complication rates of infection, seroma, hematoma, and capsular contracture as well.

Our early breast revision rate in direct-to-implant single-stage immediate breast reconstruction patients was 4,7 percent (one case), which is slightly lower than the revision rate of 28.6 percent recently reported in a small direct-to-implant single-stage immediate breast reconstruction series by Roostaeian [38]. In the largest series by Salzberg et al., an overall revision rate of 19.1 percent can be deduced by combining elective revisions (15.2 percent) with revisions resulting from complications (3.9 percent) [39]. Given that, this cohort represents our early experience using SERI®, our lower early revision rate may be attributable to the restriction of indications associated with this technique.

The main limitation of our study is the small cohort size and the absence of breasts with postoperative irradiation. Comparing our results with Gdalevitch experience [25,26], irradiation was not found to be statistically different between the successful and failed single-stage groups, likely because of the small number of irradiated patients in his cohort [32 breasts (18.9 percent)]. In a larger group of patients, widening the indications, we would expect preoperative/postoperative irradiation to be statistically significant as predictors of single-stage potential failure related, by using SERI®, on radiotherapy as well.

We are aware about our preliminary report considering small population and short follow up. We will continue to follow up our direct-to-implant single-stage immediate breast reconstruction cohort by using SERI®, for long-term revision rates to be sure about definitive results with this new surgical scaffold.

## Conclusions

Direct-to-implant single-stage immediate breast reconstruction offers many advantages to both patients and the health care system. Correctly identifying patients most likely to have a successful outcome in a single stage will allow health care providers to better allocate health care resources. In this study, we present our early experience with direct-to-implant single-stage immediate breast reconstruction by using SERI®. Patients with smaller, medium breasts had successful single-stage outcomes. The really encouraging results of our early experience will help surgeons introducing SERI® into their practice to select appropriate patients for direct-to-implant single-stage immediate breast reconstruction. In summary, the use of SERI® in implant-based reconstruction appears to be effective. A larger study cohort and longer follow-up times are required to identify additional predictors and indications.

## Consent

Written informed consent was obtained from the patient for the publication of this report and any accompanying images.
